# The Prognostic Impact of High On-Treatment Platelet Reactivity with Aspirin or ADP Receptor Antagonists: Systematic Review and Meta-Analysis

**DOI:** 10.1155/2014/610296

**Published:** 2014-10-13

**Authors:** Fabrizio D'Ascenzo, Umberto Barbero, Marta Bisi, Claudio Moretti, Pierluigi Omedè, Enrico Cerrato, Giorgio Quadri, Federico Conrotto, Giuseppe Biondi Zoccai, James J. DiNicolantonio, Mauro Gasparini, Sripal Bangalore, Fiorenzo Gaita

**Affiliations:** ^1^Department of Internal Medicine, Division of Cardiology, Città Della Salute e Della Scienza, Turin, Italy; ^2^Department of Medico-Surgical Sciences and Biotechnologies, Sapienza University of Rome, Latina, Italy; ^3^Mid America Heart Institute at Saint Luke's Hospital, Kansas City, MO, USA; ^4^Politecnico of Turin, Turin, Italy; ^5^New York University School of Medicine, New York, NY, USA

## Abstract

*Objective*. Negative results of recent randomized clinical trials testing the hypothesis of target therapy for patients with high on-treatment platelet reactivity (HOPR) have questioned its independent impact on clinical outcomes. 26 studies with 28.178 patients were included, with a median age of 66.8 (64–68) and 22.7% (22.4–27.8), of female gender. After a median follow-up of 1 year (0.1–1), cardiac adverse events occurred in 8.3% (3–11; all results are reported as median and interquartile range) of patients. Pooling all studies together, on-treatment platelet reactivity significantly increased the risk of adverse events (OR 1.33 [1.09, 1.64], *I* ^2^ = 0%). However, a sensitivity analysis showed that HOPR did not increase the risk of adverse events for patients with ACS, AMI, or stable angina as well as patients resistant to aspirin, ADP antagonists, or both. For all studies, publication bias was formally evident; after adjusting for this, HOPR did not significantly increase adverse cardiac events (OR 1.1 : 0.89–1.22, *I* ^2^ 0%). *Conclusions*. After adjusting for clinical confounders (like risk factors and clinical presentation) and for relevant publication bias, HOPR was not an independent prognostic indicator in unselected patients with both stable and unstable coronary disease for an adverse cardiac event. The clinical importance of HOPR for high-risk populations remains to be assessed.

## 1. Introduction

Aspirin and ADP receptor antagonists represent an unquestionable strategy for patients undergoing percutaneous coronary intervention (PCI), both for stable and unstable coronary disease [1]. High on-treatment platelet reactivity (HOPR), variously defined and analyzed, has been reported in up to 30% of these patients [2] and has been linked to adverse cardiac events at follow-up [3–6].

Due to the high prevalence of HOPR and the assumption that HOPR increases the risk of adverse cardiac events, randomized clinical trials were performed to test the safety and efficacy of a tailored strategy (defined as an increase in dose or a switch to another ADP receptor antagonist) in patients undergoing PCI. When appraised separately, most of these studies were negative, without achieving the expected reduction in recurrent thrombotic events [7–9].

Prognostic impact of HOPR was assessed by at least two meta-analyses, although limited from methodological flaws [3, 4], due to lack of adjustement for baseline differences in burden of traditional risk factors and clinical presentation, which may explain themselves the increased risk of adverse cardiac events in selected patients. These two studies, however, have not tested the independent clinical effect of inadequate platelet inhibition on outcomes; moreover they evaluated patients with different risk profiles (ACS and stable angina) and different treatments (aspirin together with ADP antagonists or periprocedural glycoprotein inhibitors [10, 11]).

Randomisation of patients to HOPR and non-HOPR groups is obviously not feasible; consequently a bias analysis may help to elucidate the impact of HOPR on clinical prognosis independently from cardiovascular risk factors and clinical presentations.

## 2. Methods

The recent Preferred Reporting Items for Systematic reviews and Meta-Analyses (PRISMA) amendment to the Quality of Reporting of Meta-analyses (QUOROM) statement, and recommendations from The Cochrane Collaboration and Meta-analysis of Observational Studies in Epidemiology (MOOSE) were followed during the development of the present systematic review [11–16].

### 2.1. Search Strategy and Study Selection

Pertinent articles were searched in Medline, Cochrane Library, Biomed Central, and Google Scholar in keeping with established methods with MESH strategy and with the following terms: (Prognosis/Broad[filter]) AND (platelet * AND (reactivity OR aggregation OR activation OR response *) AND (death OR (myocardial AND infarction))). Three independent reviewers (Fabrizio D'Ascenzo, Umberto Barbero, and Marta Bisi) screened the retrieved citations via the title and/or abstract; divergences were resolved via consensus. If potentially pertinent, studies were then appraised as complete reports according to the following explicit selection criteria. Studies were included if (i) reporting more than 50 patients (ii) independent prognostic impact of HOPR evaluated through multivariate analysis, while exclusion criteria were (i) nonhuman setting, (ii) duplicate reporting (in which case the manuscript reporting the largest sample of patients was selected), and (iii) interventional studies.

### 2.2. Data Extraction, End Points, and Sensitivity Analysis

Three unblinded independent reviewers (Fabrizio D'Ascenzo, Umberto Barbero, and Marta Bisi) abstracted the following data on prespecified forms: authors, journal, year of publication, location of the study group, and baseline clinical and interventional features. Data extraction was conducted by mutual agreement and all potential disagreement was solved by consensus. Incidence of adverse cardiac events (all-cause mortality and cardiovascular mortality, nonfatal myocardial infarction and stroke, and revascularization and stent thrombosis) was the primary end point. Sensitivity analyses were performed appraising aspirin and ADP receptor antagonists separately. Similarly we appraise indications for PCI in stable and unstable disease (i.e., either unstable angina, ST and non-ST segment elevation myocardial infarction). Finally, we analyze all-cause death, stent thrombosis and major bleedings.

### 2.3. Internal Validity and Quality Appraisal

Unblinded independent reviewers (Fabrizio D'Ascenzo, Umberto Barbero, and Marta Bisi) evaluated quality of included studies on prespecified forms. Modifying the MOOSE items to take into account the specific features of included studies [11], we separately abstracted and appraised study design, setting, and data source, as well as risk of analytical, selection, adjudication, detection, and attrition bias (expressed as low, moderate, or high risk of bias, as well as incomplete reporting leading to inability to ascertain the underlying risk of bias).

### 2.4. Data Analysis and Synthesis

Continuous variables are reported as mean (standard deviation) or median (interquartile). Categorical variables are expressed as *n*/*N* (%). Statistical pooling was performed according to a random-effect model with generic inverse-variance weighting, computing risk estimates with 95% confidence intervals, using RevMan 5 (The Cochrane Collaboration, The Nordic Cochrane Centre, Copenhagen, Denmark), and Comprehensive Meta-Analysis. Metaregression analysis was performed to identify impact of length of follow-up on results. Small study bias was appraised by graphical inspection of funnel plots and formally through Begg and Mazumdar rank correlation, Egger's regression intercept, and Duval and Tweedie trim and fill [14].

## 3. Results

**Figure 1:**
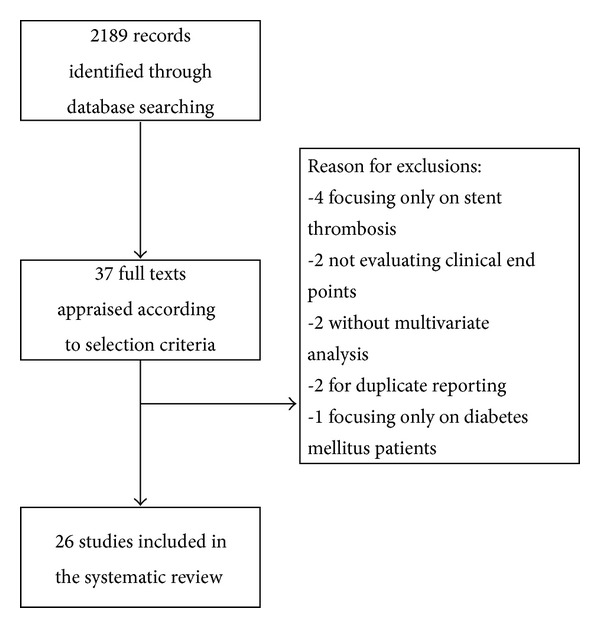
Review's profile.

**Figure 2:**
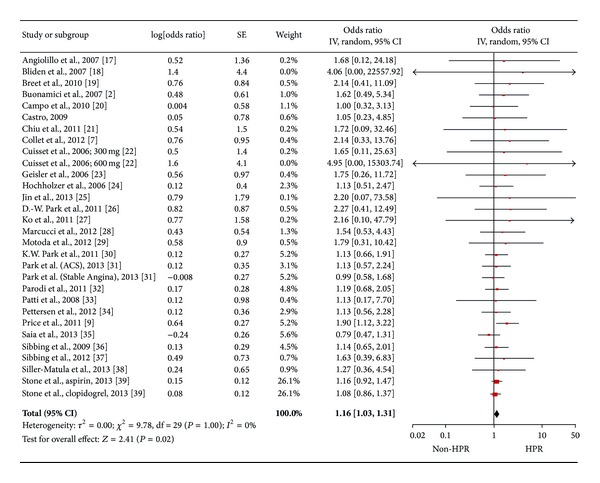
Pooled analysis of odds ratio for platelet reactivity for all studies [28.178 patients].

**Figure 3:**
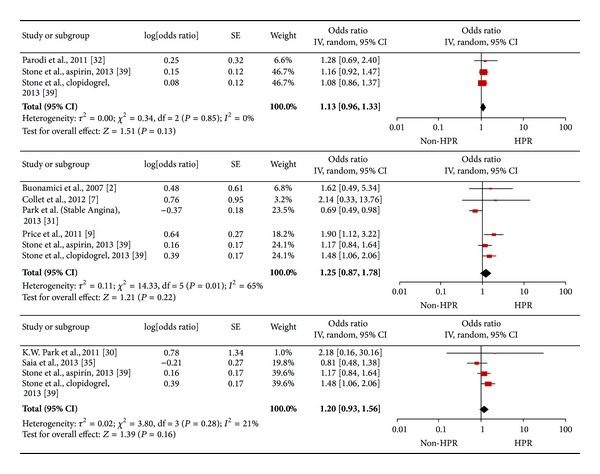
Pooled analysis of odds ratio according to end point (all-cause death [19099 patients], stent thrombosis [25848 patients] and clinically relevant bleeding [19472 patients] from above to below).

2189 records were identified through database searching, and 38 were appraised at text level and finally twenty-six studies (see Appendix) were included (Figure 1) including 28.178 patients. The median age was 66.8 (64–68), with 22.7% (22.4–27.8) being female. Diabetes mellitus, hypertension, hyperlipidemia, and a history of previous MI were reported in 29% (24.2–34), 84% (58.9–89), 70% (54.4–71), and 30% (18–39), respectively. Stable angina was the admission diagnosis for 45% (37–100) of patients, ACS for 45% (33–100), and AMI for 12% (0–34). HOPR on aspirin was reported in 25% (22–26) of population, 29% (25–37) for patients on ADP receptor antagonists, and 26% (22–39) for both (Tables 1, 2, and 3). After a median follow-up of 1 year (0.1–1), adverse cardiac events occurred in 8.3% (3–11) of patients. Pooling all studies together, HOPR significantly increased the risk of adverse cardiac events (OR 1.33 [95% CI: 1.09, 1.64], *I* ^2^ 0%, Figure 2). At metaregression analysis, length of follow up did not influence these results (Beta −0.001, *P* 0.58). HOPR did not increase risk of death (OR 1.13 [0.96, 1.33], *I* ^2^ 0%), of stent thrombosis (OR 1.25 [0.87, 1.78], *I* ^2^ 0%), and of major bleedings (1.20 [0.93, 1.56], *I* ^2^ 21%, Figure 3).

**Figure 4:**
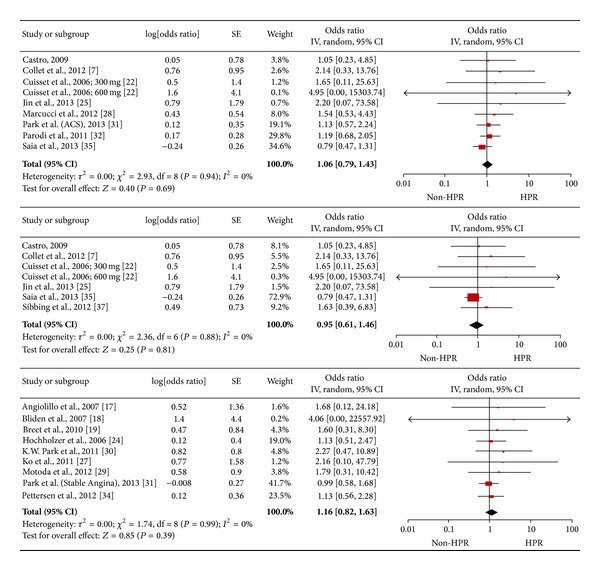
Pooled analysis of odds ratio for platelet reactivity according to diagnosis (ACS [3103 patients], acute myocardial infarction [2189 patients], stable angina [4487 patients] from above to below).

Sensitivity analysis for diagnosis showed that HOPR did not increase the risk of adverse cardiac events for patients with ACS (1.06 [0.79, 1.43], *I* ^2^ = 0%), AMI (0.95 [0.61, 1.46], *I* ^2^ = 0%), or stable angina (1.16 [0.82, 1.63], *I* ^2^ = 0%, Figure 4).

**Figure 5:**
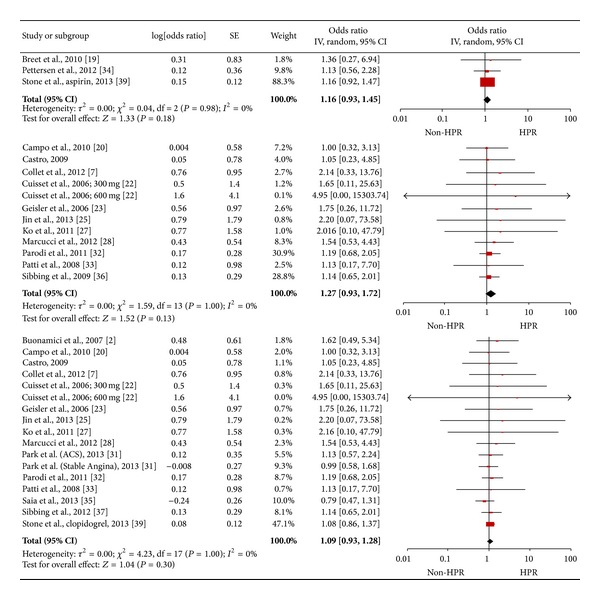
Pooled analysis of odds ratio according to reactivity (aspirin: 10066 patients; ADR receptor antagonists: 6750 patients; both: 17436 patients, from above to below in Figure 5).

Sensitivity analysis according to type of antiplatelet medication indicated that neither was HOPR an independent predictor of adverse cardiac events, nor did this show if patients were resistant to aspirin, ADP antagonists (clopidogrel in all studies), or both (1.16 [0.93, 1.45], *I* ^2^ = 0%; 1.09 [0.93, 1.28], *I* ^2^ = 0%; and 1.26 [0.70, 2.27], *I* ^2^ = 0%, Figure 5).

**Figure 6:**
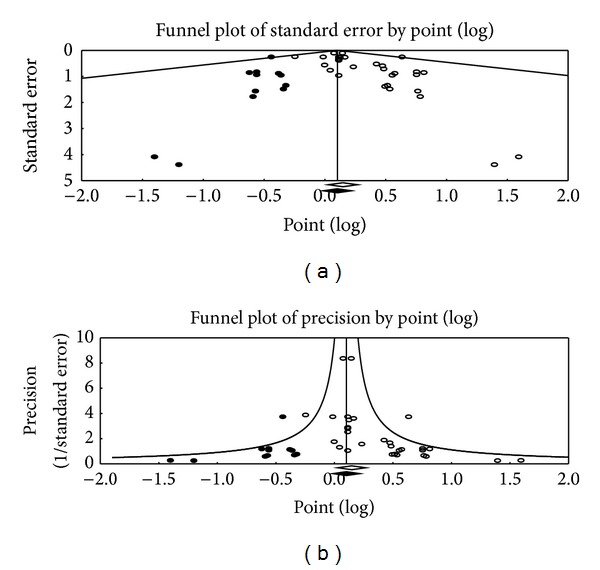
Funnel plot of standard error (a) and of precision (b). White box: observed studies. Black box: imputed study (trim and fill methods evaluates publication bias by evaluating number of “asymmetric” trials on the right side, removing and replacing them with missing counterparts at the pooled estimate and evaluating the adjusted confidence interval).

For all studies, publication bias was graphically evident (Figure 6) and formally assessed with Begg and Mazumdar rank correlation (with a positive Tau of 0.31) and with Egger's regression intercept (Intercept 0.42 : 0.11–0.69; *t*-value 2.81). After adjusting for this bias with Duval and Tweedie trim and fill, HOPR was not a significant prognostic indicator for all studies (OR 1.1 : 0.89–1.22, *I* ^2^ 0%; trim and fill methods evaluate publication bias by evaluating number of “asymmetric” trials on the right side, removing and replacing them with missing counterparts at the pooled estimate, and evaluating the adjusted confidence interval [14]).

## 4. Discussion

The main results of the present meta-analysis, investigating incidence and impact of HOPR on prognosis, are as follows: (a) HOPR represents a frequent finding for patients with coronary artery disease, both in chronic and acute settings; (b) current evidence is limited from relevant publication bias; (c) after adjustment for clinical and methodological confounders HOPR appraised for “all comers” with CAD does not significantly increase the hazard of adverse cardiac events; and (d) usefulness in high-risk patients may not be excluded and remains to be assessed.

Many reasons can explain nonresponsiveness to antiplatelet medications, such as interindividual variability in the metabolism of clopidogrel (which is a prodrug activated by CYP-3A4, CYP-2C19, and CYP1A2), drug-drug interactions (i.e., interaction on the same metabolic pathway for clopidogrel, but also competition for binding sites on COX-1 by nonsteroidal anti-inflammatory medications and aspirin), P2Y12 receptor polymorphisms and increased platelet turnover during inflammation, acute coronary events, and diabetes mellitus. Interestingly, conventional cardiovascular risk factors themselves (smoking, diabetes, and hyperlipidemia) and also the same clinical pattern of unstable angina, increasing macrophage's thromboxane synthesis, enhance resistance to aspirin [40].

Previously, numerous observational studies have demonstrated the causal relationship between laboratory evidence of nonresponsiveness to aspirin or clopidogrel and an increase hazard of death, myocardial reinfarction, and stent thrombosis during secondary prevention for coronary disease [18, 19, 23, 41–43]. The obvious induction was that individualization of antiplatelet therapy based on laboratory tests should improve outcomes, even if most of these studies were limited by absence of multivariate adjustments, that is, without a global assessment of potential clinical confounders [19], for example, the presence of diabetes, which increases both HOPR and recurrent cardiac events after ACS.

However, subsequent randomized controlled trials questioned this hypothesis. In the ARMYDA-2 study, pretreatment with a 600 mg loading dose of clopidogrel given before PCI was demonstrated to be safe and, as compared with the 300-mg dose, reduced periprocedural MI without increased bleeding [44]. On the other hand, the GRAVITAS and the ARCTIC trials, which randomized patients with HOPR after PCI with drug eluting stents to high-dose clopidogrel compared with standard-dose, did not showe significant improvements in clinical outcomes [22, 33]. Later, new evidence suggested that a more tailored therapy could be attained by switching to newer drugs [9, 45, 46]. Similarly, randomized evidence failed to demonstrate a clinical impact. The TRIGGER-PCI study showed that HOPR after elective PCI with DES implantation, if detected, can be reliably corrected by switching from clopidogrel to prasugrel but again failed to demonstrate an improvement in clinical outcomes [47]. A similar result emerged from the TRILOGY-ACS trial, randomizing patients with NSTE-ACS who were medically managed [48]. More recently, switching to ticagrelor seems to be associated to an effective reduction in HOPR but studies about the effective clinical impact are still lacking [47, 49].

This meta-analysis indicates that HOPR does not seem to be a useful predictor of outcomes in an “all comers” CAD population. These results hold true both for overall studies, and, after appraisal for diagnosis, types of antiplatelet medication analysed and assays were exploited. These findings may be explained because they derive from data drawn from multivariate analysis, with a critical adjustment (even though limited by absence of randomization itself) for clinical features both increasing platelet resistance and risk of adverse events (like diabetes mellitus, smoking, or renal disease).

While HOPR should not totally be disregarded, a focus on high-risk patients seems more appropriate [49–53], for example, those with recurrent stent thrombosis in the absence of periprocedural or adherence problems or in diabetic or in HIV populations who have a well-known increased risk of recurrent events.

Current evidence remains burdened from relevant publication bias, which deeply affects clinical interpretation of HOPR. This phenomenon was described by psychologist Robert Rosenthal as the “file drawer problem"; he wrote that “journals are filled with the 5% of the studies that show Type I errors, while the file drawers are filled with the 95% of the studies that show nonsignificant results” [54]. In the cardiovascular field, this problem was recently demonstrated by Ioannidis and colleagues [55], who stated that, among 56 meta-analyses reporting relationships between biomarkers and cardiovascular events, only 13 were not affected by selection bias. However, most of current guidelines do not include this kind of evaluation, which may deeply influence every day clinical decisions.

Our analysis has some limitations, including a great number of observational studies, which brings incomplete data around follow-up and about the correct reporting of adverse effects, different definitions, and outcomes. Moreover, for each sensitivity analysis, the number of patients was inferior to that of overall population, although superior or similar to that of previous meta-analysis on this topic [3, 4]. Again, just a small number of studies could reliably monitor compliance. Platelet reactivity tests differed in each study, which also limits the HOPR definition. Because of the selection criteria, no studies selected use the Platelet Vasodilator-Stimulated Phosphorylation test (PLT-VASP test), a flow cytometry test that is today the most specific test to assess the effect of the platelet P2Y12 antagonists (clopidogrel, ticlopidine, and prasugrel) [51]. Thus, the included studies' quality was evaluated according to standardized criteria and we separately abstracted and appraised study design, setting, and data source, as well as risk of analytical, selection, adjudication, detection, and attrition bias. For all studies, publication bias was formally assessed. After adjusting for this bias, HOPR did not significantly increase adverse cardiac events for all studies.

We therefore conclude that routine assessment of HOPR is not useful, but high-risk subsets of patients (i.e., diabetics, multiple cardiovascular risk factors, and important comorbidities, especially if they need therapies potentially interacting with antiplatelet drugs) may potentially benefit from its assessment and interpretations remain to be assessed.

## Figures and Tables

**Table 1:** Baseline features of included studies.

	Number of patients	Age	Female patients (%)	Diabetes mellitus (%)	Hypertension (%)	Hyperlipidemia (%)	Previous myocardial infarction (%)	Stable angina (%)	Acute coronary syndromes (%)	Myocardial infarction (%)
Angiolillo et al., 2007 [17]	173	67 ± 9	35	100	65	68	53	100	0	0
Bliden et al., 2007 [18]	100	66 ± 11	28	44	74	83	40	75	13	12
Breet et al., 2010 [19]	410	64 ± 11.3	26.8	17.3	72.4	77.6	58.3	100	0	0
Breet et al., 2010 [19]	920	64 ± 10.6	24.6	18.4	77.5	80.9	54.6	100	0	0
Buonamici et al., 2007 [2]	804	69 ± 11	25	21	62	50	26	34	39	27
Campo et al., 2010 [20]	826	68 ± 12	25.2	24	72.3	59.4	38.6	64.4	35.6 (low risk UA)	—
Chiu et al., 2011 [21]	144	65 ± 10	24	46.5	68.8	50	18	55	45	—
Collet et al., 2012 [7]	106	64 ± 10	23	25	58	56	46 (previous ACS)	0	100	0
Cuisset et al., 2006; 300 mg [22]	146	64.2 ± 10.3	21	29	58	56	44 (previous ACS)	0	100	100
Cuisset et al., 2006; 600 mg [22]	146	65.2 ± 12	27	33	56	55	45 (previous ACS)	0	100	100
Geisler et al., 2006 [23]	379	67.5 ± 10	26.9	34.7	79.6	60.6	45.5 (previous ACS)	54	45	—
Hochholzer et al., 2006 [24]	802	66.4 ± 9.1	21.8	24.8	82.3	nd	22.9	100	0	0
Jin et al., 2013 [25]	181	61.3 ± 12.1	16.6	24.9	39.5	95.5	3.9	0	100	100
D.-W. Park et al., 2011 [26]	809	64	33.2	30.5	66.3	45.4	7.2	100	—	—
Ko et al., 2011 [27]	222	63.3	31.5	32.0	72.1	46.8	5.9	100	—	—
Marcucci et al., 2012 [28]	1187	69	25.2	24.0	65.4	54.4	x	—	100	35
Motoda et al., 2012 [29]	450	71.1	31.5	42.8	74.0	60.2	31.1	100	—	—
K. W. Park et al., 2011 [30]	2546	61.7	29.9	28.5	58.9	61.0	5.9	55.6	44.4	—
**Park et al. (ACS), 2013 [31]**	**1095**	**62**	**21**	**26**	**60**	**58**	**5**	**—**	**100**	
**Park et al. (Stable Angina), 2013 [31]**	**1329**	**63**	**22**	**27**	**57**	**63**	**4**	**100**	**0**	**0**
Parodi et al., 2011 [32]	1789	69	20	19.8	57.0	44.7	18.1	—	100	46
Patti et al., 2008 [33]	160	66	19.3	34.3	nd	74.3	28.1	45.7	54.3	—
Pettersen et al., 2012 [34]	1001	62.3	21.8	20.0	55.4	98.3	43.7	100	—	—
Price et al., 2011 [9]	380	68	23.2	28.9	88.2	35.5	31.6	100	—	—
Saia et al., 2013 [35]	833	67.6	25	28.7	69.3	67.5	32.0	0	0	100
Sibbing et al., 2009 [36]	1608	67.5	23.0	29.0	91.6	70.0	32.0	66.9	33	20
Sibbing et al., 2012 [37]	564	67.7	22.3	31.2	89.3	70.5	19.1	—	100	100
Siller-Matula et al., 2013 [38]	403	64.2	24.1	32.0	84.6	76.4	32.0	67	33.0	33.0
**Stone et al., 2013 [39]**	**8665**	**63.6**	**26**	**32.4**	**79.6**	**74.3**		**48.3**	**27.6**	**24.1**

**Table 2:** Incidence of reactivity on aspirin, clopidogrel or both and kind of assays used.

	Reactivity on aspirin and ADP receptor antagonists (%)	Reactivity on aspirin (%)	Reactivity on ADP receptor antagonists (%)	Assays used
Angiolillo et al., 2007 [17]	—	—	**25**	Light Transmittance Aggregometry (ADP 20 mmol/L-upper quartile)
Bliden et al., 2007 [18]	—	—	22 (LTA) 30 (TEG)	Light Transmittance Aggregometry (ADP 5 mmol/L) Thromboelastography
Breet et al., 2010 [19]	14.7	8.5 (aspirin only)	25.1 (clopidogrel only)	Verify Now aspirin/Verify Now P2Y12
Breet et al., 2010 [19]	26.9 (LTA 5) 23.3 (LTA 20)	21.1 (LTA 5) 24.7 (LTA 20)	14.9 (LTA 5) 13.0 (LTA 20)	Light Transmittance Aggregometry (ADP 5 mmol/L-LTA 5, and 20 mmol/L-LTA 20)
Buonamici et al., 2007 [2]	—	—	13	Light Transmittance Aggregometry (ADP 10 mmol/L)
Campo et al., 2010 [20]	3	15	21.6	Verify Now aspirin/Verify Now P2Y12
Chiu et al., 2011 [21]	—	—	33	Platelet Function Analyzer-100
Collet et al., 2012 [7]	—	27	26	Both ADP and arachidonic acid (AA) as agonists to explore the responses to clopidogrel and aspirin, respectively
Cuisset et al., 2006; 300 mg [22]	—	—	25	Light Transmittance Aggregometry (ADP 10 mmol/L)
Cuisset et al., 2006; 600 mg [22]	—	—	15	Light Transmittance Aggregometry (ADP 10 mmol/L)
Geisler et al., 2006 [23]	—	—	5.8	Light Transmittance Aggregometry (ADP 20 mmol/L)
Hochholzer et al., 2006 [24]	—	—	50	Verify Now P2Y 12
Jin et al., 2013 [25]	—	nd	55	Multiple electrode aggregometry, Verify Now P2Y 12, Verify Now Aspirin
D.-W. Park et al., 2011 [26]	—	—	40.9	Light Transmittance Aggregometry (ADP 10 mmol/L)
Ko et al., 2011 [27]	52	—	—	ADP-induced platelet aggregation using a whole blood analyzer
Marcucci et al., 2012 [28]	11	17	44	Multiple electrode aggregometry
Motoda et al., 2012 [29]	—	—	50	Multiple electrode aggregometry
K. W. Park et al., 2011 [30]	—	—	25	Verify Now P2Y 12
**Park et al. (ACS), 2013 [31]**			**63**	Verify Now P2Y 12
**Park et al. (Stable Angina), 2013 [31]**			**61**	Verify Now P2Y 12
Parodi et al., 2011 [32]	—	26	—	PFA 100
Patti et al., 2008 [33]	—	—	32.1	Verify Now P2Y 12
Pettersen et al., 2012 [34]	—	—	20	Multiple electrode aggregometry (ADP)
Price et al., 2011 [9]	—	—	36	Multiple electrode aggregometry (ADP)
**Saia et al., 2013 [35]**	**—**	**—**	**67**	**Verify Now P2Y12**
Sibbing et al., 2009 [36]	8	27	19	Multiple electrode aggregometry (AA and ADP)
Sibbing et al., 2012 [37]				
Siller-Matula et al., 2013 [38]				
**Stone et al., 2013 [39]**			**42.7**	**Verify Now aspirin/Verify Now P2Y12**

**Table 3:** Incidence and definition of outcome appraised in the multivariate model.

	Follow-up (months)	Definition of outcome	Incidence of outcome
Angiolillo et al., 2007 [17]	24	Cardiovascular death, ACS, and stroke	15.2 1st quartile
			12.2 2nd quartile
			12.2 3rd quartile
			37.7 4th quartile

Bliden et al., 2007 [18]	1 12	Death secondary to any cardiovascular cause, stroke, myocardial infarction (ami), and target/nontarget vessel revascularization	23 (1 month FU) 50 (12 months FU)

Breet et al., 2010 [19]	12	All-cause death, nonfatal ami, stent thrombosis, and stroke	LTA 5 11.3 (DHPR)
			8.8 (HAPR)
			10.9 (HCPR)
			4.1 (NPR)
			LTA 20 10.7 (DHPR)
			9.6 (HAPR)
			11.7 (HCPR)
			4.2 (NPR)

Buonamici et al., 2007 [2]	6	Stent thrombosis	3.1

Campo et al., 2010 [20]	12	All-cause death, nonfatal ami, and stroke	Full Responder (FR) 8.6
			Poor Responder (PR) 15.8
			ASA FR 10 PR 13
			Clop FR 5.9 PR 17.3

Chiu et al., 2011 [21]	24	Cardiovascular death, nonfatal myocardial infarction, or nonfatal stroke	10

Collet et al., 2012 [7]	1	Stent thrombosis	2

Cuisset et al., 2006; 300 mg [22]	1	Cardiovascular death, nonfatal ami, stent thrombosis, and stroke	12
			33.3 HPR 0.5 NPR

Cuisset et al., 2006; 600 mg [22]	1	Cardiovascular death, nonfatal ami, stent thrombosis, and stroke	4.1
			27.2 HPR 0.008 NPR

Geisler et al., 2006 [23]	3	Cardiovascular death, nonfatal ami, and nonfatal stroke	6.6
			5.6 Adequate clopidogrel response
			22.7 Low clopidogrel response

Hochholzer et al., 2006 [24]	1	All-cause death, nonfatal ami, and percutaneous revascularization	1.9
			3.5 in upper quartile

Jin et al., 2013 [25]	12	Cardiovascular death, nonfatal ami, and nonfatal stroke	11

D.-W. Park et al., 2011 [26]	12	Cardiac death and nonfatal ami	1.4
			0.9 Adequate clopidogrel response
			2.8 Low clopidogrel response

Ko et al., 2011 [27]	1	All-cause death, nonfatal ami, nonfatal stroke, and percutaneous revascularization	8.6

Marcucci et al., 2012 [28]	12	Cardiac death and nonfatal ami	9.6

Motoda et al., 2012 [29]	12	Cardiac death, nonfatal ami, stent thrombosis, and target vessel revascularization	12
			19 in HPR
			5.1 in NPR

K. W. Park et al., 2011 [30]	24	Cardiac death, nonfatal ami, nonfatal stroke, and urgent percutaneous revascularization	14.6 HPR
			8.7 LPR

**Park et al. (ACS), 2013 [31]**	72	Cardiac death, nonfatal ami, nonfatal stroke, urgent percutaneous revascularization, and stent thrombosis	

**Park et al. (Stable Angina), 2013 [31]**	72	Cardiac death, nonfatal ami, nonfatal stroke, urgent percutaneous revascularization, and stent thrombosis	

Parodi et al., 2011 [32]	1	Cardiac death, nonfatal ami and percutaneous revascularization	3 1st quartile
			5 2nd quartile
			10 3rd quartile
			20 4th quartile

Patti et al., 2008 [33]	24	All-cause death, nonfatal ami, unstable angina, and stroke	13.3 HAPR
			9.9 LAPR

Pettersen et al., 2012 [34]	6	Cardiovascular death, nonfatal myocardial infarction, and stent thrombosis	6.5 HPR
			1 LPR

Price et al., 2011 [9]	1	Stent thrombosis	2.2 HPR
			0.2 LPR

**Saia et al., 2013 [35]**	**12**	**All-cause death, ami, and urgent target vessel revascularization**	

Sibbing et al., 2009 [36]	1	All-cause death, ami, and urgent target vessel revascularization	Abciximab/UFH: 9.4 HPR 6.7 LPR
			Bivalirudin: 22.0 HPR 5.0 LPR

Sibbing et al., 2012 [37]	12	Acute coronary syndrome, stent thrombosis, stroke, death, and revascularization	37.5 DHPR
			33.3 HCPR
			25.6 HAPR
			18.6 LPR

Siller-Matula et al., 2013 [38]		Acute coronary syndrome, stent thrombosis, stroke, death, and revascularization	

**Stone et al., 2013 [39]**	**24**	**All-cause death and myocardial infarction and stent thrombosis**	**2.4 death**
			**3.9 mi**
			**1.3 ST**

## Abbreviations

ACS: Acute coronary syndrome
AMI: Acute myocardial infarction
HOPR: High on-treatment platelet reactivity
PCI: Percutaneous coronary intervention.
